# Clinical and conventional pharmacy services in Polish hospitals: a national survey

**DOI:** 10.1007/s11096-015-0234-9

**Published:** 2016-01-06

**Authors:** Iga Pawłowska, Leszek Pawłowski, Ivan Kocić, Natalia Krzyżaniak

**Affiliations:** Department of Pharmacology, Medical University of Gdańsk, Dębowa Str. 23, 80-204 Gdańsk, Poland; Department of Palliative Medicine, Medical University of Gdańsk, Dębinki Str. 2, 80-211 Gdańsk, Poland; Department of Pharmacy, Graduate School of Health, University of Technology Sydney, 638 Jones Street, Broadway, Australia

**Keywords:** Clinical pharmacy, Clinical services, Conventional services, Hospital pharmacy, Pharmaceutical services, Pharmacist, Poland

## Abstract

*Background* Pharmacist-led care services within the hospital pharmacy setting have a significant impact on efficient drug management processes. The work of pharmacists is directly associated with the provision of drugs and medical supplies along with additional clinical, administrative, organizational and educational duties. Depending on the country, these practice roles may differ to a significant extent. *Objective* The aim of this research was to explore the role of the hospital pharmacist and the provision of both clinical and traditional pharmaceutical services for patients and medical staff in Polish general hospitals. *Setting* Hospital pharmacies from all general hospitals in Poland. *Method* A cross-sectional study was conducted, utilizing an anonymous questionnaire as the research instrument. Heads of hospital pharmacies were requested to participate in this study and complete the questionnaire. The survey was initially piloted to improve the research method. *Main outcome measure* The types of pharmaceutical services performed in Polish general hospitals. *Results* 166 hospital pharmacies took part in this survey. The overall response rate was 60.8 %. The total number of full-time equivalent (FTE) professionals employed within the surveyed hospital pharmacies was approximately 833. The procurement and distribution of drugs were identified as pharmaceutical services performed by most of the participants. The significant majority of pharmacists were also involved in compounding, adverse drug reaction monitoring and rational drug management services. Eleven (7 %) of the responding pharmacists had direct contact with patients and 7 (4 %) pharmacists took part in ward rounds. More precise legal regulations regarding hospital pharmacy practice were measures indicated by most pharmacists as necessary changes required in the hospital pharmacy system. *Conclusion* Polish hospital pharmacists provide various pharmaceutical services. Their work is closely related with direct provision of drugs. There is an observed inadequate level of clinical services provided in comparison to clinical settings in other countries.

**Electronic supplementary material:**

The online version of this article (doi:10.1007/s11096-015-0234-9) contains supplementary material, which is available to authorized users.

## Impact on practice

- The majority of the pharmaceutical services provided by hospitals pharmacists are directly associated with medicines, their production, procurement, distribution and storage.
- In comparison to other countries, patient oriented pharmaceutical services are not a common practice within Polish general hospitals.
- More precise legal regulations and an increase in staffing levels within the pharmacy may improve the quality of services in Polish hospital pharmacy.

## Introduction

Pharmacist-led care services within the hospital pharmacy setting have a significant impact on efficient drug management processes. According to the European Association of Hospital Pharmacists (EAHP), the fundamental role of the hospital pharmacy service is to “optimize patient outcomes, by collaboratively working within multidisciplinary teams in order to achieve responsible use of medicines” [1]. In order to promote quality patient care, a hospital pharmacist’s role comprises of the provision of both clinical pharmacy services and conventional pharmacy services i.e. the dispensing and production of medicines [2]. According to the European Society of Clinical Pharmacy, the clinical pharmacist is responsible for performing activities focusing on the promotion of rational and appropriate use of medicinal products and devices [3]. However, depending on the country, the extent to which these listed roles are performed in practice may differ. For example, clinical services are more commonly performed in the US than in Europe. In Europe, only 6 % of pharmacists carry out services on wards for a minimum of 50 % of their total work time, whilst in the US 34 % of hospital pharmacists spend more than 8 h per day on the ward. Differences are also observed between particular countries in Europe; in the UK, Ireland and Norway clinical pharmacy practice is highly developed in comparison to their counterparts in Eastern Europe [4]. Evidence from other countries confirms that clinical pharmacy services performed within the following units: intensive care [5, 6], geriatric [7], oncology [8], internal medicine [8, 10], pediatrics [11], surgery [12, 13], cardiology [14] and palliative care [15], are beneficial for patients, hospital staff and the health care system. Pharmacists are also essentially involved in the detection and prevention of harmful medication errors [16–20] and drug related-problems, including adverse drug reactions [21–23]. Gerdemann et al. in their pilot study demonstrated that the presence of pharmacy interns on the ward increased drug safety by detecting potential prescribing and documentation errors (e.g. inappropriate drug or its dosage, drugs interactions) as well as improving drug storage conditions on the ward. Furthermore, the interns ward attendance and recommendations resulted in a high rate of acceptance among physicians and nurses at the examined German hospital [24]. Apart from providing clinical services, the fundamental purpose of hospital pharmacy is to efficiently and safely supply medicines. Since the cost of medicines ranges from 5 to 12 % of total hospital expenditures in developed countries, appropriate drug selection and procurement is essential in the rationalization of therapy, for which pharmacists are responsible [25]. Additionally, pharmacists must ensure the adequate distribution, storage, safety and quality of all medicines at the hospital. At present, 40 % of US hospitals operate on a decentralized medication use-system and 58 % of hospitals plan to use it in the future [26]. Data highlights that in comparison to previous years, there is an observed growth in adapting decentralized models [27, 28]. In contrast, only 6.5 % of hospitals in Europe use a decentralized distribution model, with 70 % utilizing a centralized system. However Europe is reporting similar tendencies as the US in converting to the decentralized model of practice [29, 30]. In conjunction with distribution and procurement procedures, medicine production and compounding is another valuable service performed by the hospital pharmacy. The production of sterile and non-sterile products in Europe is performed by 44 and 67.5 % of hospital pharmacies respectively. However, the compounding of products has seen a considerable decrease in recent years, dropping from 71 and 92.5 %, for sterile and non-sterile products respectively from 2000 [30]. Similarly to other countries, Polish hospital pharmacists frequently perform professional activities to ensure the safe and efficient use of drugs. It is a requirement that these pharmacy services are performed in accordance with Polish law (Pharmaceutical Law and Pharmaceutical Chambers’ Act) and each hospital’s own rules and regulations. The services provided by hospital pharmacy specifically pertain to the use of pharmacotherapy, and involve drug supply, compounding, clinical activities, patient safety, administrative duties, education as well as scientific research. A preliminary study conducted at Polish teaching hospitals demonstrated that all of the Polish pharmacists surveyed were solely responsible for the ordering and dispensing of medicines. A significant number of pharmacists were also actively involved in the compounding of medicines, collaborating in drug management processes at the hospital as well as participating in clinical trials. However, it is reported that patient oriented clinical activities at teaching hospitals were rarely performed with 94 % of pharmacists failing to participate in hospital medical rounds, and 44 % claiming to have never worked in the capacity of a clinical pharmacist [31].

### Aim of the study

The aim of this research was to explore the clinical role of the hospital pharmacist and the associated clinical and conventional pharmacy services performed within the hospital setting. The study seeks to further investigate the level of collaboration between pharmacists and other medical staff and to identify areas of potential service improvement in order to understand the necessary future changes that need to be introduced to Polish general hospital practice.

### Ethical approval

The pharmacists are not the direct subjects of this study, as we have only examined their opinions on current hospital pharmacy practice. The responses to the survey were anonymous and all respondents agreed to participate in the study by completing and returning a written questionnaire.

## Method

A cross sectional study was carried out to collect survey data from a selected population of hospital pharmacists. The research was performed on a national scale; the heads of each pharmacy department employed at every general hospital (teaching and non-teaching) in Poland were recruited to participate in this research. The total number of participants was 273. They were chosen from the widely accessible web database of Health Care Institutions (accessed 2013). The questionnaire was developed based on legislation regulating pharmacist services and hospital pharmacy practice, literature and the researchers own experience. The survey was anonymous. It was initially piloted with just the Polish teaching hospitals and corrected following the feedback [31]. The questionnaire consisted of both multiple-choice questions and open-ended questions (the questionnaire is available as a supplementary material). The multiple-choice group made up the majority of questions in the survey, and enabled the collection of reliable and uniform data and the further application of statistical methods. The questionnaire along with a letter of invitation, (which included information about the survey and a request for participation in this research), was distributed by Polish Post to predetermined hospitals. The respondents, as representatives of all professional pharmacy staff employed at their respective hospital, were asked to fill-in the questionnaire and return it in the enclosed envelope by post or by fax or email. The survey was conducted in 2013.

Subsequent to receiving the results, the Chi square test and Fisher’s exact test (for small groups) as well as descriptive statistics were applied to the data. The Chi square test was applied to test whether the variables (population of the city in which the hospital is located or the number of professionals employed at the pharmacy) are associated with the performance frequency of particular pharmaceutical services. Additionally, we have used this test to examine the relationship between the age of pharmacists and the willingness to introduce any changes into the hospital pharmacy system. Furthermore, we tested if the size of the hospital had any influence on the provision of pharmaceutical care, and whether the level of pharmacist experience had any impact on collaborative practice with other medical staff. Descriptive statistics were used to summarize the data and show the basic characteristics of the population. All statistical analyses were calculated with the use of STATISTICA Version 10 software (StatSoft, Inc., 2010).

## Results

166 out of 273 hospitals took part in this survey (response rate 60.8 %). Most of the respondents were employed by large hospitals, characterized by facilities containing more than 100 beds. Out of the 166 hospitals that were included in the study, a total number of 833 full-time equivalent (FTE) professionals were found to be employed in their respective pharmacies; with an average of 5 ± 4.94 FTE per pharmacy (median = 4, the range extended from 1 to 39 FTE per pharmacy) (Tables 1, 2).

**Table 1:** Background characteristics of the surveyed hospitals

	n = 166	%
*Number of beds*		
≥10 ≤ 30	1	1
>30 ≤ 60	2	1
>60 ≤ 100	8	5
>100	154	93
No response	1	1

			Average per pharmacy
*The number of full-time equivalent (FTE) professionals employed in hospital pharmacies*			
Total	833.4	100	5
PhD, pharmacist	9	1	0.05
Master of pharmacy	372.9	45	2.2
Pharmacy technician	451.5	54	2.7

**Table 2:** Background characteristics of the heads of hospital pharmacies

	n = 166	%
*Age*		
>30 years ≤ 40 years	34	20
>40 years ≤ 50 years	45	27
>50 years ≤ 60 years	61	37
>60 years	26	16
*Gender*		
Female	137	83
Male	28	17
No response	1	1
*Education*		
PhD, pharmacist	5	3
Master of pharmacy	160	96
No response	1	1
*Specialization*		
Clinical pharmacy	6	4
Hospital pharmacy	41	25
Community pharmacy	111	67
Other	8	5
*During the course (not completed)*	17	10
Clinical pharmacy	5	3
Hospital pharmacy	7	4
Community pharmacy	3	2
Lack of specialization	26	16
*Length of service to the hospital pharmacy*		
<1 year	2	1
≥1 year ≤ 3 years	10	6
>3 years ≤ 10 years	42	25
>10 years	112	67
*Direct supervisor*		
Hospital director	75	45
Vice director	87	52
Other	4	2

Table 3 details the types of pharmaceutical services performed by pharmacists and highlights the differences in service according to the size of the cities in which the hospitals are located (number of inhabitants/population) and the FTE of professional staff in the pharmacies.

**Table 3:** Types of pharmaceutical services performed at hospital pharmacies in context of the population of the cities in which the hospitals are located and the number of hospital pharmacy staff

	Service	Service provided	Number of pharmacies in context of the population of the city in which the hospital is located		Number of pharmacies in context of the full-time equivalent (FTE) professionals	
			<50,000 (n = 91)	>50,000 (n = 72)	≤3 (n = 76)	>3 (n = 88)
1.	Dispensing of drugs and medical devices	166 (100 %)	91	72	76	88
2.	Procurement of drugs and medical devices from warehouses	160 (96 %)	88	69	73	85
3.	Co-participation in the management of drugs at the hospital	159 (96 %)	86	70	73	84
4.	Giving information about action and indication of drugs and medical devices	153 (92 %)	81	69	69	82
5.	Compounding	144 (87 %)	77	64	*57*	*86*
6.	Co-participation in the rationalization of therapy	117 (70 %)	64	50	50	65
7.	Co-participation in adverse drug reaction monitoring	110 (66 %)	*52*	*55*	46	63
8.	Co-participation in clinical trials at the hospital	60 (36 %)	*15*	*43*	*11*	*49*
9.	Preparing drugs, including cytotoxics in daily doses	33 (20 %)	*9*	*23*	*4*	*29*
10.	Preparing total parenteral nutrition	20 (12 %)	*6*	*14*	*4*	*16*
11.	Preparing solutions for hemodialysis and peritoneal dialysis	4 (2 %)	*0*	*4*	0	4
12.	Preparing solutions for enteral nutrition	2 (1 %)	0	2	0	2
13.	Preparing iv solutions	2 (1 %)	1	1	1	1

Italic numbers describe the statistically significant differences within variables in each of the two groups (*p* < 0.05)

The concept of pharmaceutical care was incorporated into Polish legislation in 2008 and is defined as a documented practice in which a pharmacist together with the patient, physician and other healthcare professionals ensures the appropriate use of pharmacotherapy in order to achieve positive clinical outcomes that increase the patients quality of life. As such, this service, as defined by the aforementioned law, was performed by only 17 out of 166 hospital pharmacies (10 %). The provision of this relatively new service is observed more often at smaller hospitals (5 settings out of 11) than in bigger settings (12 out of 15) (*p* = 0.00008). The respondents listed two main reasons for the poor rates of involvement in pharmaceutical care, including: lack of time due to an insufficient number of pharmacy staff (32, 19 %) and lack of perceived need for this service from a physicians’ perspective (22, 13 %). All of the surveyed pharmacists were found to carry out their work within hospital dispensaries. Only a small number of pharmacists additionally performed services on hospital wards and in drug units (15, 2 % respectively). At 162 general hospitals (98 %), the pharmacist was found to be a member of a Drug and Therapeutics Committee and a further 22 pharmacists declared to be the president or vice president of the committee.

**Fig. 1:**
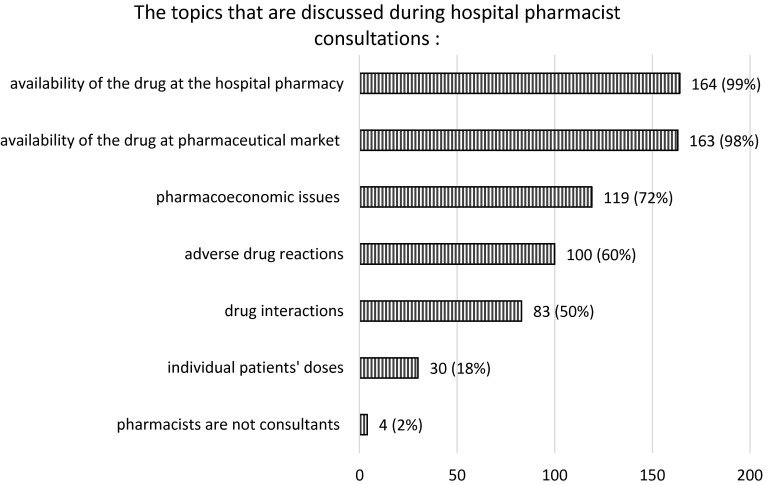
The topics that are discussed during hospital pharmacist consultations

Pharmacists were most often found to collaborate professionally with the head nurse (151, 91 %), the hospital director (120, 72 %) and the head of the ward (115, 69 %). A smaller number of pharmacists claimed to work in partnership with on–call physicians (39, 23 %) and other staff (medical director, administrative staff, microbiologist, physician, and clinical pharmacologist), (32, 19 %). The main topics of pharmacist consultations with these staff members are presented in Fig. 1.

Pharmacists with a period of service greater than 10 years within the hospital reported more often that they collaborated with other medical staff than less experienced workers (*p* < 0.005), (e.g. 106 out of 112 pharmacists with work experience longer than 10 years collaborate with a head nurse, whereas 45 out of 53 less experienced workers collaborate with a head nurse). Clinical pharmacologists were found to play a minor role at Polish general hospitals, with only 6 out of 166 (4 %) facilities employing this type of specialist, and only 3 respondents collaborating with clinical pharmacologists. In terms of the clinical services provided, only 11 out of 166 pharmacists (7 %) reported to having direct contact with patients. According to respondents, these interactions occur during the dispensing of medicines in special drug programs, during chemotherapy as well as during outpatient counseling following specialist medical consultations. Patient counseling by pharmacists occurred more commonly within hospitals located in bigger cities (9 out of 71), than at facilities with smaller populations (2 out of 91), (*p* = 0.009, Fisher’s exact test). 154 (93 %) of the surveyed Polish hospital pharmacists do not participate in multidisciplinary ward rounds. Only 7 (4 %) pharmacists admitted to taking part in ward rounds, with 2 respondents participating on a regular basis and 5 occasionally. Despite this finding, 54 (33 %) heads of pharmacy reported that they would like to change the situation and become involved in ward rounds. However 100 (60 %) pharmacists were not interested in undertaking this activity. 108 (65 %) respondents subjectively believed that pharmacists employed within the hospital pharmacy were not clinical pharmacists at all and only 3 pharmacists (2 %) were certain that they fulfilled their services as a clinical pharmacist. Other participants claimed that their clinical role was rather negligible.

**Fig. 2:**
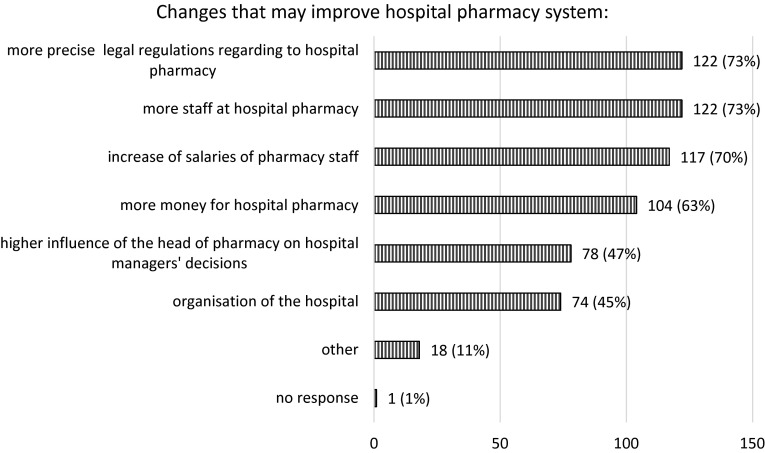
Changes that may improve hospital pharmacy system according to the pharmacists

The final section of the survey considers proposals for change from the respondent’s perspective, which should be introduced into the hospital pharmacy system to improve pharmaceutical services. According to 73 (44 %) pharmacists, changes to the current system are necessary and 67 (40 %) respondents determined that only minor alterations ought to be made. Alternatively, 22 (13 %) participants believe that there is no need for any amendments to current hospital pharmacy practice. Younger pharmacists (<50 years of age) are more interested in introducing changes (45 out 78) than older pharmacists (28 out 74), (*p* = 0.005). As Fig. 2 shows, more precise legal regulations of the hospital pharmacy system, increased levels of staff within pharmacies as well as salary increases were commonly identified by the majority of respondents as important measures to implement to improve practice. Pharmacists also listed a number of other changes perceived as being essential in order to advance current practice. These included: (1) the development of modern and advanced hospital pharmacy equipment, e.g. systems unit dose, (2) implementing changes to pharmacist tertiary education, i.e. the emphasis of the study curriculum should be shifted from chemical structures to more clinical issues, (3) providing extra postgraduate education options for career progression, as well as the (4) employment of staff holding specialist qualifications and undertaking further education (clinical pharmacists, pharmacologists).

## Discussion

To our knowledge, this is the first time a nationwide study of this nature has been conducted in our country. A key reason for undertaking this study was to understand the necessary future changes needed in Polish hospital pharmacy that will lead to the increase of delivering quality pharmaceutical services as well as optimizing patient outcomes. The presented study was performed on a large scale—a complete sample of objectives participated in this research—the heads of hospital pharmacies employed in each general hospital in the whole of Poland were invited to participate. Therefore, the findings collected from 60 % of all general hospitals present a description of the current status of pharmaceutical service performance within hospitals in our country. Furthermore, in comparison to other similar studies utilizing questionnaires, the observed proportion of returned surveys is satisfactory [26, 32, 33].

Firstly, a significant number of the investigated hospitals are large clinical settings that in total, employ more than eight hundred specialized workers, including: doctors of pharmacy, pharmacists and pharmacy technicians, whose activities are described in this study. In regards to the pharmaceutical services provided by pharmacists, the supply of medicines for inpatients was provided by the majority of the pharmacies investigated. This responsibility may be considered as being one of the elemental tasks of a pharmacist’s work within the hospital. Additionally, a number of hospital pharmacies were involved in drug production and compounding (144/160, 87 %). It is assumed that the calculated figure encompassed both sterile and non-sterile products. A 2010 study conducted by EAHP highlighted that in Poland, the production of non-sterile drug formulations for inpatients was performed at 85.7 % of pharmacies whereas sterile production was less common [30]. Thus the obtained result is comparable with the mentioned survey. In comparison to Europe, where the average production level of non-sterile medicines is 65.8 % and decreasing, the Polish level of compounding is high [30]. Furthermore, the advanced services as e.g. co-participation in clinical trials, preparing total parenteral nutrition as well as the preparation of drugs, including cytotoxics, in daily doses are more often performed at hospitals located in big cities than in their counterparts located in smaller cities. Additionally, it was seen that the performance of these services was more common at pharmacies employing a larger number of professional staff.

In general, the results of the study indicate that clinical practice in Polish hospital pharmacies is not highly developed. With reference to patient-oriented activities, only 10 % of pharmacists admitted to carrying out pharmaceutical care services, with 15 % stating that they worked on the hospital wards. Only 4 % of respondents indicated that they take part in hospital ward rounds, with two pharmacists out of the entire study population claiming to participate in rounds on a regular basis. Direct contact with patients was initiated by a small minority of participants, and three pharmacists out of 166 regarded themselves as being clinical pharmacists. Pharmacists were found to collaborate most often with the head nurse or the hospital director, which indicates that these consultations may involve administrative or organizational issues instead of clinical ones. In light of these findings the following question arises: why are clinical services not considered to be a priority in Polish general hospitals as is observed in other countries [4]? This problem is complex and requires a comprehensive approach. Firstly, the majority of pharmacists are not sufficiently equipped or prepared to offer clinical services. Whilst the current Polish curriculum of pharmacy degrees prepares graduates for work within both hospital and community pharmacies, the majority of young pharmacists will gain employment and perform pharmacy services within the latter setting. Therefore, pharmacy courses are more focused on the preparation of graduates for work within community pharmacies, with training involving for example, classes held at virtual training pharmacies, where students learn how to cope with patients in real community pharmacies [34]. Furthermore, during the 6th year of study, 960 h (6 months) of practice are required to be performed at a community pharmacy, where students learn how to dispense prescription and non-prescription drugs, counsel patients, perform administrative tasks and prepare drugs. According to the law, in some cases students may have to perform a portion of this practice (not more than 3 months) within hospital pharmacy settings. However, it is not mandatory, and most candidates do not have chance to gain such experience at a hospital pharmacy. On the other hand, article 44 of the EU Directive 2005/36/EC deals that the six month period of training may be undertaken in “a pharmacy that is open to the public or in a hospital” [35]. In addition to the students being unable to experience hospital pharmacy practice, as the study indicates, most of the pharmacists who actually work within hospital pharmacies do not have a clinical specialization. Another issue is that some pharmacists are not willing to engage in clinical activities i.e. they do not want to take part in a hospital ward rounds and are not interested in changing the current situation. Unquestionably, clinical services should be carried out in direct contact with both patients and medical staff [36, 37]. The data highlighted that the conditions for collaboration between pharmacists and other medical staff within Polish settings are not favorable, as only a small number of pharmacists perform pharmaceutical services on hospital wards. Lack of time, associated with a shortage of pharmacy staff, overwhelming levels of duties and a perceived lack of need for pharmaceutical services were pointed out by hospital managers as the reasons for poor engagement in clinical activities. One positive aspect, evidenced in the results, emphasizes that Polish pharmacists work together with other medical staff to improve the delivery of health care, through active participation in Drug and Therapeutic Committees, which are also established in other countries [38, 39]. A survey by Tan et al. [38] indicated that senior medical staff and hospital pharmacists comprise the core of these committees. Additionally, in some cases, Polish pharmacists are presidents or vice-presidents of this group. These pharmacist attitudes indicate their interest and engagement in rational drug usage and hospital drug policy.

Finally, the study collected data regarding the respondents’ perspectives on future changes in hospital pharmacy. It is encouraging, that the majority of hospital managers’, particularly younger representatives, believe that the Polish hospital pharmacy system requires remodeling. In the opinion of 44 % of respondents, these changes should be extensive. Most of the hospital managers indicated that changes in legislation, (that should be implemented into hospital pharmacy law), may improve the performance of pharmaceutical services. Furthermore, they detailed that an increase in staffing levels within the pharmacy, (which was also mentioned as a condition), may increase the provision of quality pharmaceutical care. This opinion may result from that fact that pharmacists currently feel that they are being overwhelmed by duties that include administrative work and documentation, which are responsibilities that are not directly associated with clinical pharmacotherapy. It is worth mentioning, that pharmacists are also implicated in the economic aspects of medicine usage, and there is a significant amount of evidence that suggests pharmacist-initiated interventions have a positive influence on decreasing therapy costs [40–42].

### Study limitation

The results of the survey should be discussed in the context of some limitations. Firstly, the questionnaire was not administered orally, but was sent by post. The respondents could have potentially misunderstood some questions, and did not have a chance to clarify them with the researcher. Next, the questionnaire was not detailed and occupied one page A4, therefore some questions were not extensively examined. However, the questionnaire was intentionally designed to be short in format to increase the willingness and participation of respondents to complete and return it and obtain a satisfactory response rate.

## Conclusion

In conclusion, these findings provide a preview into the current state of clinical and traditional Polish hospital pharmacy. In general, hospital pharmacy is more developed in bigger cities and within pharmacies employing a larger number of pharmacists. In comparison to other countries, clinical services in Polish settings should be more commonly performed. However, some single examples of patient oriented activities in hospital pharmacy have been observed. A large number of Polish pharmacists are involved in activities directly associated with drug management including compounding, supply of medicines or participation in Drug and Therapeutic Committees. There are a variety of adjustments that have been proposed which may improve Polish hospital pharmacy. The most important were changes in law related to hospital pharmacy practice and increased financing.

Further research focusing on the opinions of other healthcare providers (physicians, nurses) on the quality of pharmaceutical services is required to provide a comprehensive insight into the current state of Polish hospital pharmacy practice. This, in turn, improves understanding on the necessary changes that should be introduced to hospital pharmacy practice.

## Electronic supplementary material

Below is the link to the electronic supplementary material.

Supplementary material 1 (DOCX 57 kb)
