# Supplementary material for: Clinical and conventional pharmacy services in Polish hospitals: a national survey
Source: Int J Clin Pharm. 2016 Jan 6;38:271–9. doi: 10.1007/s11096-015-0234-9 (PMC4828469; doi:10.1007/s11096-015-0234-9)
Supplement: Supplementary file 1 — Supplementary material 1 (DOCX 57 kb) [file 11096_2015_234_MOESM1_ESM.docx]

**An anonymous survey conducted among the heads of hospital pharmacies in multi-specialist hospitals in Poland - 2013**

| **Background characteristics of the hospital pharmacy** | |
| --- | --- |
| **1. The number of hospital beds that are serviced by the hospital pharmacy:**  A)  < 10  B)  ≥ 10 ≤ 30  C)  > 30 ≤ 60  D)  > 60 ≤ 100  E)  > 100 | **2. The number of professional staff working within the hospital pharmacy (including the pharmacy manager):**  A) PhD in Pharmacy……………….  B) Master of Pharmacy……………...  C) Pharmacy technicians……….... |

| **Background characteristics of the heads of hospital pharmacies** | | |
| --- | --- | --- |
| **3. Age:**  A)  ≥ 25 ≤ 30 years of age  B)  > 30 ≤ 40 years of age  C)  > 40 ≤ 50 years of age  D)  > 50 ≤ 60 years of age  E)  > 60 years of age | **4. Gender:**  A)  female  B)  male | **5. Level of education:**  A)  PhD in Pharmacy  B)  Master of Pharmacy |
| **6. Specialisation:**  A)  Clinical Pharmacy  B)  Hospital Pharmacy  C)  Community Pharmacy  D)  Other *(Describe)*……………………  E)  In the process of completing specialisation *(Describe)*………………  F)  No specialisation | **7. Years of experience in hospital pharmacy practice:**  A)  < 1 year  B)  ≥ 1 ≤ 3 years  C)  > 3 ≤ 10 years  D)  >10 years | **8. Who is the immediate supervisor to the head of the hospital pharmacy?**  **……………………………………** |

| **Information about the services and activities carried out by the hospital pharmacy - current status** |
| --- |
| **9. Types of pharmacy services performed by the hospital pharmacy:** *(possible to select multiple answers)*  A)  dispensing of drugs and medical devices for wards  B)  compouding  C)  preparing total parenteral nutrition  D)  preparing solutions for enteral nutrition  E)  preparation of daily doses of medications, including cytotoxics  F)  preparation of radiopharmaceuticals for the needs of patients of a given diagnosis  G)  preparing  *iv* solutions  H)  procurement of drugs and medical devices from warehouses  I)  preparing solutions for haemodialysis and peritoneal dialysis  J)  preparation of other prescription-based medications  K)  co-participation in monitoring adverse drug reactions  L)  co-participation in clinical trials at the hospital  M)  co-participation in the rationalisation of therapy  N)  co-participation in the management of drugs at the hospital  O)  giving information about action and indication of drugs and medical devices |
| **10. Is pharmaceutical care provided by the hospital pharmacy understood as a documented practice in which a pharmacist together with the patient, physician and other healthcare professionals ensures the appropriate use of pharmacotherapy in order to achieve positive clinical outcomes that increase the patients quality of life?**  A)  yes B)  no (*why*?)....................................................................... |
| **11. Pharmacists perform their duties:** *(possible to select multiple answers)*  A)  in the hospital pharmacy B)  on hospital wards C)  other location, describe........................ |
| **12. Pharmacists perform a consultative role for doctors and other medical staff, consisting of providing information on:**  *(possible to select multiple answers)*  A)  the availability of the drug at the pharmaceutical market  B)  the availability of the drug at the hospital pharmacy  C)  adverse drug reactions  D)  drug interactions  E)  individual patients’ doses  F)  pharmacoeconomic issues  G)  pharmacists are not consultants |
| **13. Are pharmacists members of the Drug and Therapeutic Committee?**  A)  yes *(what role do they perform? – chairman, member – important to describe)*  B)  no |
| **14. The pharmacist works primarily with:**  A)  director of the hospital  B)  head of ward  C)  on-call physician  D)  head nurse  E)  other staff member………………………  F)  pharmacist does not work with other staff |
| **15. Is there a clinical pharmacologist employed in the hospital?**  A)  yes, (*do they work together with pharmacists?)*………………………………………………….  B)  no |
| **16. Do pharmacists participate in daily rounds on any of the wards of the hospital?**  A)  yes, often  B)  yes, occasionally  C)  no and not willing to participate  D)  no, but would like to participate |
| **17. Does the pharmacist have direct contact with patients?**  A)  yes, *(to what extent?)* …………………………………………………………..  B)  no |
| **18. Is research conducted by the pharmacy? (excluding pharmacoeconomic studies)**  A)  yes, *(to what extent?)*……………………………………………………………………  B)  no |
| **19. Does the pharmacy conduct pharmacoeconomic research?**  A)  yes, *(to what extent?)*…………………………………………………………………………  B)  no |
| **20. What percentage of pharmacists roles are based on providing clinical pharmacy services?**  A)  100% B)  75% C)  50% D)  25% E)  0% |
| **21. What services are performed in order to lower the costs of therapy?** *(possible to select multiple answers***)**  A)  pharmacoeconomic analyses  B)  negotiating with the wholesalers supplying the hospital  C)  negotiating with pharmaceutical companies  D)  restricting the purchasing of medications  E)  obtaining donations of drugs or medical supplies from pharmaceutical companies  F)  offering tenders for the purchase of drugs or medical supplies  G)  participation of the hospital in clinical research  H)  purchasing cheaper, bioequivalent medications  I)  none of the above |
| **22. What percentage of pharmacy needs are fulfilled by funds from the national health fund (NFZ) given to the hospital pharmacy for the purchase of medications and medical devices?**  A)  100% B)  75% C)  50% D)  25% E)  0% |

| **Information concerning the future needs of the hospital pharmacy** |
| --- |
| **23. Is there a need to change pharmacist roles within the hospital pharmacy?**  A)  yes B)  yes, but not to a great extent C)  no |
| **24. Is there a need for greater pharmacist participation in directly consulting patients about their pharmacotherapies?**  A)  yes B)  no |
| **35. What kind of changes would improve the hospital pharmacy system?** *(possible to select multiple answers)*  A)  implementing more precise legal regulations regarding to hospital pharmacy practice  B)  more money for the pharmacy  C)  increase of salaries of pharmacy staff  D)  organisational changes within the hospital  E)  increasing the influence of the head of pharmacy’s opinions on the hospital manager’s decisions  F)  more staff at hospital pharmacy  G)  other……………………………………………………………………………………………….  ……………………………………………………………………………………………………… |

**Would you like to receive the results from the research?..........................Please provide an e-mail address for the results to be sent to………………………………………………………..**
